# A Precise Method to Evaluate 360 Degree Measures of Optic Cup and Disc Morphology in an African American Cohort and Its Genetic Applications

**DOI:** 10.3390/genes12121961

**Published:** 2021-12-09

**Authors:** Victoria Addis, Min Chen, Richard Zorger, Rebecca Salowe, Ebenezer Daniel, Roy Lee, Maxwell Pistilli, Jinpeng Gao, Maureen G. Maguire, Lilian Chan, Harini V. Gudiseva, Selam Zenebe-Gete, Sayaka Merriam, Eli J. Smith, Revell Martin, Candace Parker Ostroff, James C. Gee, Qi N. Cui, Eydie Miller-Ellis, Joan M. O’Brien, Prithvi S. Sankar

**Affiliations:** 1 Scheie Eye Institute, University of Pennsylvania, Philadelphia, PA 19104, USA; Victoria.Addis@pennmedicine.upenn.edu (V.A.); Rebecca.Salowe@pennmedicine.upenn.edu (R.S.); ebdaniel@pennmedicine.upenn.edu (E.D.); roylee@pennmedicine.upenn.edu (R.L.); pistilli@gmail.com (M.P.); Jinpeng.Gao@Pennmedicine.upenn.edu (J.G.); maguirem@pennmedicine.upenn.edu (M.G.M.); Lilian.Chan@pennmedicine.upenn.edu (L.C.); gudiseva@pennmedicine.upenn.edu (H.V.G.); Selam.Zenebe-Gete@pennmedicine.upenn.edu (S.Z.-G.); sayakam@seas.upenn.edu (S.M.); smiteli@pennmedicine.upenn.edu (E.J.S.); ermartin@pennmedicine.upenn.edu (R.M.); cparker@pennmedicine.upenn.edu (C.P.O.); Qi.Cui@pennmedicine.upenn.edu (Q.N.C.); Eydie.Miller@pennmedicine.upenn.edu (E.M.-E.); Prithvi.Sankar@pennmedicine.upenn.edu (P.S.S.); 2 Department of Radiology, University of Pennsylvania, Philadelphia, PA 19104, USA; minchen1@upenn.edu (M.C.); gee@upenn.edu (J.C.G.); 3 Penn Vision Research Center, University of Pennsylvania, Philadelphia, PA 19104, USA; zorger@pennmedicine.upenn.edu

**Keywords:** optic nerve, stereo disc images, glaucoma, cup-to-disc ratio, African Americans, genetic associations with cup-to-disc ratio

## Abstract

(1) Background: Vertical cup-to-disc ratio (CDR) is an important measure for evaluating damage to the optic nerve head (ONH) in glaucoma patients. However, this measure often does not fully capture the irregular cupping observed in glaucomatous nerves. We developed and evaluated a method to measure cup-to-disc ratio (CDR) at all 360 degrees of the ONH. (2) Methods: Non-physician graders from the Scheie Reading Center outlined the cup and disc on digital stereo color disc images from African American patients enrolled in the Primary Open-Angle African American Glaucoma Genetics (POAAGG) study. After converting the resultant coordinates into polar representation, the CDR at each 360-degree location of the ONH was obtained. We compared grader VCDR values with clinical VCDR values, using Spearman correlation analysis, and validated significant genetic associations with clinical VCDR, using grader VCDR values. (3) Results: Graders delineated outlines of the cup contour and disc boundaries twice in each of 1815 stereo disc images. For both cases and controls, the mean CDR was highest at the horizontal bisector, particularly in the temporal region, as compared to other degree locations. There was a good correlation between grader CDR at the vertical bisector and clinical VCDR (Spearman Correlation OD: r = 0.78 [95% CI: 0.76–0.79]). An SNP in the *MPDZ* gene, associated with clinical VCDR in a prior genome-wide association study, showed a significant association with grader VCDR (*p* = 0.01) and grader CDR area ratio (*p* = 0.02). (4) Conclusions: The CDR of both glaucomatous and non-glaucomatous eyes varies by degree location, with the highest measurements in the temporal region of the eye. This method can be useful for capturing innate eccentric ONH morphology, tracking disease progression, and identifying genetic associations.

## 1. Introduction

Glaucoma is a progressive optic neuropathy that is characterized by retinal ganglion cell damage and resultant irreversible visual-field loss [1,2]. One of the earliest signs of glaucoma is damage to the optic nerve [3,4,5,6,7]. This damage typically presents as cupping or excavation of the optic disc, as well as thinning of the neuroretinal rim, resulting in an increased cup-to-disc ratio (CDR). CDR is most commonly defined clinically as the ratio of the vertical cup diameter to the vertical disc diameter [8]. Though an enlarged vertical CDR (VCDR) is not sufficient to diagnose glaucoma, changes in this value do correspond to death of retinal ganglion cells and, thus, serve as an important measure for evaluating the optic nerve head (ONH) and monitoring glaucoma progression [9]. 

Typically, VCDR is measured in a clinical setting by estimation and/or by describing or graphically depicting abnormalities. This method, though efficient in a clinical setting, has several limitations. First, it requires an ophthalmologist to make a one-dimensional linear estimate of the three-dimensional ONH [10]. Such a depiction—and the resultant VCDR—may not fully capture the eccentric or irregular cupping and neuroretinal rim notching seen frequently in glaucoma patients [10,11,12]. Additionally, optic-disc grading has been shown to have significant variability among ophthalmologists [9,13,14,15,16,17] and glaucoma specialists [18,19,20]. The use of trained graders can help to improve inter-observer agreement [21,22], but may overestimate agreement in the clinical setting, due to ideal conditions, such as using discs with a confirmed glaucoma diagnosis or having unlimited time to grade images [17]. 

In recent years, automated imaging of the ONH has been increasingly used to help diagnose and monitor glaucoma [23]. Among its benefits, automated imaging is more accessible to clinicians who are not glaucoma specialists [24] and makes mass screenings more feasible [25]. It can also provide alternative measures of assessing cup and disc changes in addition to VCDR, including neuroretinal rim thickness and Bruch’s membrane opening minimum rim width. Instruments used for automated optic-disc evaluation include the Cirrus high-definition optical coherence tomography (HD-OCT); the Heidelberg Retina Tomograph (HRT), a scannin g laser ophthalmoscope with two classification algorithms (glaucoma probability score [26] and Moorfields regression analysis [27]); and the KOWA Nonmyd WX 3D fundus camera, a simultaneous stereo photography device. These instruments classify glaucoma moderately well [24], with one study reporting that HRT algorithms had higher diagnostic accuracy than ophthalmologists when classifying stereo disc photos [17]. However, these tests do not always report VCDR with accuracy [28]. One study found that measurements of VCDR from the HD-OCT and HRT had poor correlation with clinical grading among a cohort of Chinese patients [28]. These instruments are also limited by their predominantly Caucasian normative databases, which may hinder disc classification for other ethnic groups with different normative ranges [28]. 

Researchers have also developed algorithms that directly compute CDR from retinal fundus images [25,29,30,31,32]. Advantages of this approach include a quick processing time and independence from normative databases. However, these algorithms often do not fully capture the eccentric morphology of the ONH, due to inconsistent segmentation of cup and disc boundaries or interference from other pathologies such as peripapillary atrophy. The resultant CDR values may not yet be robust or consistent enough for research use [33]. 

Though the above approaches have frequently been used in the diagnosis and monitoring of glaucoma patients, they are not optimal for research studies on glaucoma. Research studies could benefit from an approach that precisely measures the uneven cup and disc boundaries in glaucomatous eyes, resulting in accurate CDR values at every degree location [10,11,12]. Such an approach could be used to more accurately depict the nuances of the optic disc and to allow for closer analysis of glaucomatous progression. Additionally, this precise phenotyping will better allow for the discovery of genetic variants associated with disc structure. 

To address this need, we developed a method to measure CDR at one-degree intervals from stereo-disc images. This method uses outlines of the cup and disc boundaries from trained graders in an ophthalmology reader center to generate coordinate locations, which are used to calculate CDR at all 360 degrees of the ONH. In this paper, we introduce this method and compare its output to traditional VCDR in a large population of African Americans with and without glaucoma. We also test if three previously discovered genetic associations with clinical VCDR can be replicated by using grader VCDR measures.

## 2. Materials and Methods

### 2.1. Study Population

This study uses the color stereo-disc images of patients enrolled in the Primary Open-Angle African American Glaucoma Genetics (POAAGG) study. The POAAGG study investigates the genetics of primary open-angle glaucoma (POAG) in the overaffected African American population. The study population consists of self-identified Blacks (African American, Afro-Caribbean, or African descent) aged 35 years or older who were recruited from Philadelphia. Exclusion criteria are detailed elsewhere [34]. Every subject was classified as a case, control, or suspect by fellowship-trained glaucoma specialists based on previously published criteria [34]. The University of Pennsylvania Institutional Review Board approved this study and the informed consent process, and the research adhered to the tenets of the Declaration of Helsinki. 

### 2.2. Outlining of Cup and Disc Boundaries

This study analyzed 30-degree color stereo-disc photos (*n* = 1815) that were taken by using the Topcon TRC 50EX retinal camera (Topcon Corp. of America, Paramus, NJ, USA), from glaucoma cases, controls, and suspects. These images were received by the Ophthalmology Reading Center at the University of Pennsylvania between 26 February 2016 and 28 February 2018, through a secure uploading system. 

Three non-physician graders were trained by two fellowship-trained glaucoma specialists to grade digital stereo color images of the optic disc and to use the stereo viewer (Screen-Vu stereoscope, Portland, OR, USA). Details on training sessions are provided elsewhere [22]. 

The graders, masked to all clinical details, outlined structures on each optic nerve photograph, using the Image J/Fiji software (available at http://rsbweb.nih.gov/ij/, accessed on 1 July 2018); Rasband WS, ImageJ, US National Institutes of Health, Bethesda, MD, USA, 1997e2012). Outlined structures for this study included the following:

(1) The optic cup, using only contour and vascular cues (“contour cup”);
(2) The optic disc, defined as the outer border of the nerve rim and the inner border of the scleral ring, if a scleral ring was present.

Each stereo-disc photo was outlined by two graders. The height and width of these measurements were then calculated by using the Image J/Fiji (https://imagej.net/Fiji, accessed on 1 July 2018) software. The software calculated the height and width based on the vertical and horizontal axes of the photo, where the origin of the axes was set as the upper left corner of the photo. Region of interest (ROI) files that contained the coordinates of the outlines were saved for each image (i.e., two sets of ROIs per image).

### 2.3. Adjudication of Images

Variations can exist in the ROIs from two graders for the same image, so an adjudication procedure was created. The dice coefficients for both the disc and the cup contour were determined by using the following formula: ([2* area overlap of graders 1 and 2]/[area of grader 1 + area of grader 2]). The difference between the VCDR of each image was also compared between the two graders. When the dice coefficient was below 0.7, or the difference of VCDR was greater than 0.2 between graders, the image was sent to adjudication for further review. A total of 382 images required adjudication for this study.

During adjudication, an ophthalmologist first evaluated the original stereo-disc photo to determine if it was gradable or not (i.e., blurred, inadequate levels of stereo, etc.). If these characteristics were present to the extent that they affected grader outlines, the image was classified as ungradable and removed from the study.

For all gradable images, the ophthalmologist assessed the ROIs from the two graders. If one of the outlines was determined to be close to what the ophthalmologist would have drawn, the ROI from that grader was used in further analysis. If the ROIs from both graders were distinctly different from what the ophthalmologist would have drawn, then the ophthalmologist created a new set of outlines to use in further analysis. 

### 2.4. Polar Representation of Cup and Disc Boundaries 

**Figure 1:**
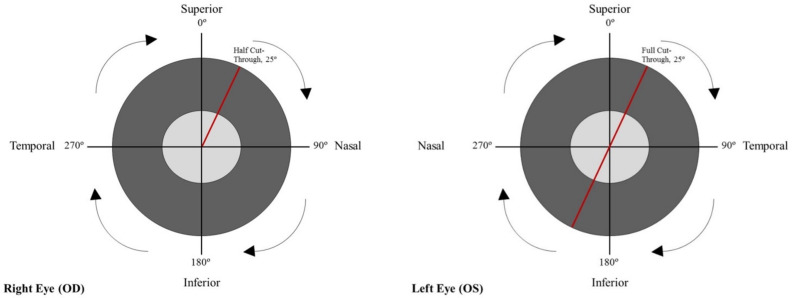
Orientation of degrees in the optic nerve head of right and left eye.

The coordinates of the cup and disc outlines from each ROI were imported into MATLAB (R2020a, https://www.mathworks.com/, accessed on 1 July 2018). The centroid of each disc was calculated by closing gaps in the outline drawings; converting outlines to binary images, using MATLAB’s *poly2mask* function; and using the *regionprops* function to determine the centroid. The coordinates of each outline were then converted from Cartesian to polar representation, with the polar origin set at the disc centroid. The 12 o’clock position was set as 0° and increased in a clockwise direction (i.e., 3 o’clock position was 90°, 6 o’clock was 180°, and 9 o’clock was 270°) for both eyes (Figure 1).

### 2.5. Half Cut-Throughs and Full Cut-Throughs

The distance from the centroid to the cup and disc boundaries was calculated at each degree. We refer to these measurements as “half cut-throughs” (instead of “radius”), due to the non-circular shape of the ONH. (The term “radius” is defined as a straight line from the center to the circumference of a circle or sphere.) The “full cut-throughs” were calculated by adding together two complementary angled half cut-throughs (Figure 1). For example, summing the half cut-through at 0° and 180° yielded the full cut-through for the vertical bisector. CDRs were then calculated as the ratio of the cut-through for the cup to the cut-through for the disc at each degree location. 

There were two sets of half cut-throughs and full cut-throughs for each stereo-disc photo from outlines drawn by each of the two graders. These values were averaged together, at each degree, for each stereo-disc photo. The only exception was for an adjudicated image where the ophthalmologist determined that the outlines of one grader or the ophthalmologist were more accurate (see above section). OD and OS were averaged separately.

**Figure 2:**
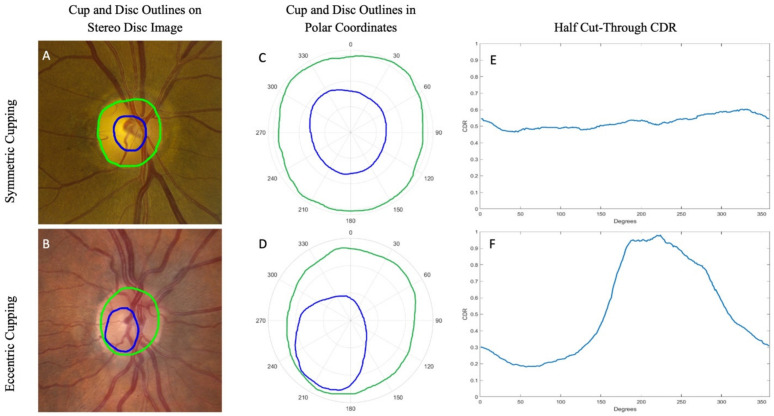
Examples of outlined stereo disc photos converted into polar coordinates for eyes (OD) with symmetric and eccentric cups, both from Bin 6 (0.5 ≤ CDR < 0.6). The stereo disc photos of patients with symmetric cupping (**A**) and eccentric cupping (**B**) are shown, both with a CDR in Bin 6 and outlined for cup contour (blue) and disc (green). The same contours were converted into polar coordinates (**C**,**D**), and the half cut-through CDRs at each degree location for these images were plotted (**E**,**F**).

The stereo-disc images were divided into ten bins, by 0.1 increments of their CDR calculated at the vertical meridian. The mean and standard deviation of the half cut-throughs and full cut-throughs were plotted at each degree for each bin. The mean and standard deviation of full and half cut-through measurements for case and control groups were compared. Polar plots were also created that averaged the outline coordinates at each degree location for each bin. Figure 2 provides two examples, illustrating the original stereo disc image (A, B), resultant polar plot (C, D), and half cut-through at each degree (E, F) for a patient with symmetric cupping and a patient with eccentric cupping.

### 2.6. Notching

Notching can be an early sign of glaucomatous damage and has a positive predictive value for glaucoma [35]. We defined notching as well-defined areas of thinning or complete loss of the rim with the extension of the cup in one localized area. Using this definition, graders qualitatively identified images with notching. The degree location of the maximum half cut-through was identified for each image. We then examined the CDR measurements in the 50 degrees surrounding this location for images with notches versus without notches.

### 2.7. Correlation with Clinical VCDR Values

The clinical VCDR was calculated by an ophthalmologist after direct observation of the patient at the slit lamp. A total of 855 subjects had available VCDR values in their electronic medical records, corresponding to 1613 stereo disc images (813 OD and 800 OS). The value closest to the date of the first stereo disc image was selected for each patient. These values were compared with the CDR at the vertical bisector calculated by using the grader outlines, using Spearman correlation analysis. 

### 2.8. Correlation with Genetic Findings 

The POAAGG study previously conducted a genome-wide association study (GWAS) on glaucoma-associated quantitative traits, including clinical VCDR, in 5049 cases and controls. This analysis identified 30 variants associated with clinical VCDR (data not shown) [36]. Of these 30 variants, three SNPs were selected for further analysis in this paper based on their allele frequency and/or prior association with POAG phenotypes. The three SNPs are located in the *CXCR7/ACKR3* (*Chr* 2:237653539, rs12328841), *MPDZ* (*Chr 9:13173885*, rs4740546), and *ADAM12* (*Chr* 10:127738557) genes. The SNPs in the *CXCR7/ACKR3* and *ADAM12* genes were chosen due to their high allele frequencies (0.41 and 0.42, respectively), and the SNP in the *MPDZ* gene was chosen due to its prior association with central corneal thickness [37]. These SNPs were tested for association with the grader VCDR and the grader CDR area ratio in 623 cases, using a generalized linear model, with adjustment by age, gender, and inter-eye correlation, using the generalized estimating equation [38,39].

## 3. Results

### 3.1. CDR Analysis

A total of 1815 stereo disc images were analyzed in this study, including eyes from 1384 cases, 316 controls, and 115 suspects. A previous study demonstrated high inter-grader reliability when non-physician graders outlined the cup and disc by using both color and vascular cues [22]. 

**Figure 3:**
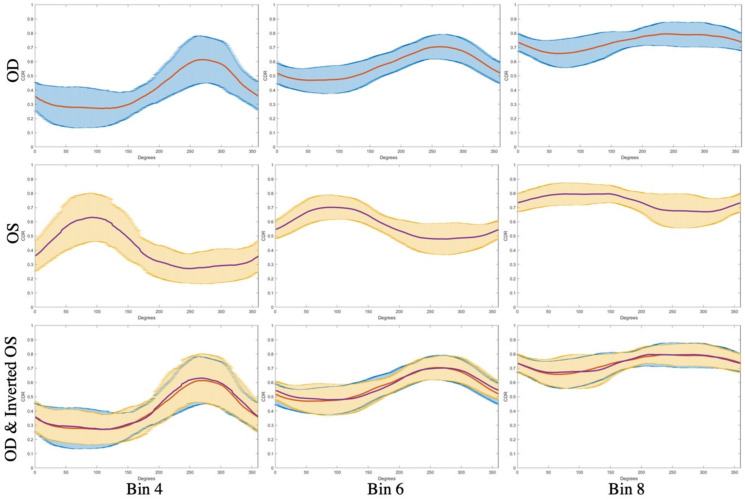
Mean (orange and purple) and standard deviation (blue and yellow) of the half cut-through CDR at each degree location of stereo disc photos for OD (top row) and OS (middle row) eyes from patients in Bins 4, 6, and 8. The bottom row shows the correspondence between the OD and OS half-cut-through pattern when the OS eyes are inverted horizontally.

When plotting the half cut-throughs for eyes, we found that the CDR varied by degree location (Figure 3), with the highest amount of variation seen in eyes with a smaller CDR (i.e., Bin 4). The mean CDR was highest in the temporal region (i.e., near 270° for OD and near 90° for OS) and lowest in the nasal region (i.e., near 90° for OD and near 270° for OS). By overlaying OD with OS (inverted horizontally), we confirmed that both the left and right eyes had identical trends; as a result, we combined OD and OS in the remainder of the analyses. 

The full cut-throughs showed a similar pattern, with the highest mean CDR observed at the horizontal bisector (i.e., 90° and 270°) and the lowest at the vertical bisector (0° and 180°) (Supplementary Materials Figure S1). The symmetry between plotted values from 0° to 180° and from 180° to 360° was expected, as these values were identical. 

**Figure 4:**
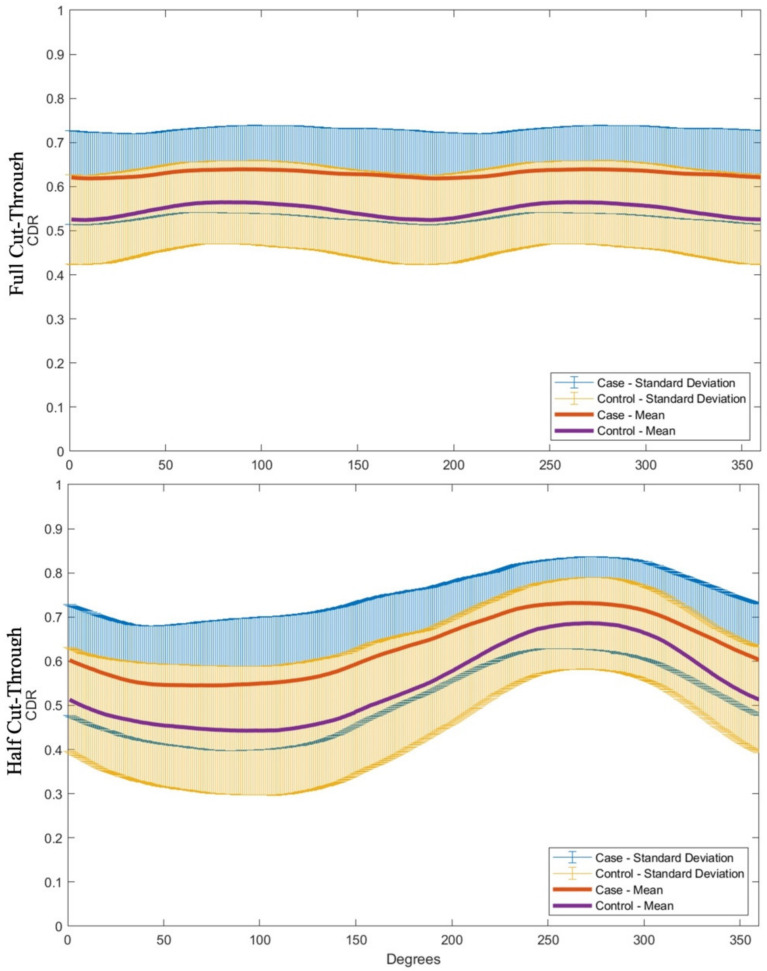
Comparison of the mean (and standard deviation) of full and half cut-through measurements between the case (N = 845) and control (N = 244) cohorts in Bins 2–8. Measurements include both OD and OS eyes from each bin, with the measurements from the OS eyes horizontally inverted to match the OD nasal/temporal directions.

A polar representation of averaged CDRs at each degree location showed the same trend, with highest CDR again in the temporal region (Supplementary Materials Figure S2). This same trend was also observed when dividing eyes by case and control groups, though the case group had a higher mean CDR than the control group, as expected (Figure 4).

We also compared eyes with and without notches by examining the 50 degrees surrounding the location of the maximum half cut-through (Supplementary Materials Figure S3). Eyes with notches had a steeper drop in CDR in the surrounding degrees, as one would expect clinically. 

**Figure 5:**
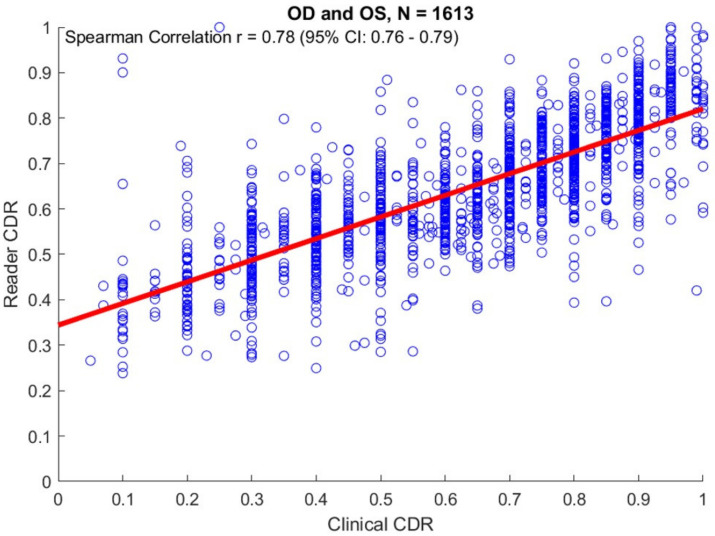
Correlation of vertical CDR measured by the trained readers versus clinicians, N = 161.

A comparison of clinical CDR and grader CDR at the vertical bisector is shown in Figure 5. There was good correlation between grader CDR at the vertical bisector and clinical VCDR (Spearman Correlation OD: r = 0.78 [95% CI: 0.76–0.79].

### 3.2. Genetic Associations

For the genetic associations, the SNP in the *MPDZ* gene showed significant association in grader VCDR (*p* = 0.01) and grader CDR area ratio (*p* = 0.02) (Table 1). The other two SNPs did not show significant associations with the grader VCDR values.

## 4. Discussion

This study introduced a precise method to measure the CDR at all 360 degrees of the ONH. The approach provides deeper insight into the eccentric morphology of the optic nerve in glaucoma, which can be used to define multiple phenotypes. Future studies can examine if these phenotypes are associated with specific genetic variants. 

This approach was successfully applied to a heterogeneous group of patients, including glaucoma cases and controls. These patients had VCDR values ranging from <0.1 to >0.9, as is consistent with prior reports that even healthy individuals show wide variation in optic cup and disc size [40,41] and broad phenotypic heterogeneity [11,12]. The morphology and shape of cups and discs also varied widely, with some being concentric (Figure 2A) and others quite eccentric (Figure 2B). These differences are well summarized by the line graph of the half cut-through values (Figure 2E,F). A line with a slope of 0 (flat line) corresponds to a perfectly concentric cup, while eyes with eccentric cupping have graphs that deviate from a flat line, with areas of greater cupping represented with high values. It is also important to note that all stereo disc images assessed in this study came from African Americans, who are at highest risk for POAG [42] and often present with more severe disease than other ethnic groups [43]. An accurate analysis of the ONH is thus especially important for this overaffected population. 

Within this clinically heterogeneous cohort, our method quantitatively showed that VCDR is often not reflective of the CDR at the other 360 degrees of the ONH in both the cases and healthy controls. The trend seen in our cohort was a lower CDR at the vertical bisector and a higher CDR at the horizontal bisector. In particular, we observed the highest CDR values in the temporal region of both eyes, as is consistent with the ISNT rule, which states that the neuroretinal rim width decreases in the order inferior (I) > superior (S) > nasal (N) > temporal (T) [44]. This trend was true not only for glaucomatous eyes, but also for healthy controls without a glaucoma diagnosis. This suggests that relying solely on VCDR values to detect or track glaucomatous damage may lead to the underestimation of cupping in other locations, such as the temporal region. Finally, this method may also be able to identify notching, which can be an important early indicator of glaucoma. As expected clinically, images with notches showed a sharper decline in CDR values in the surrounding degrees. 

We expected that our method would show a moderate correlation with VCDR values from the clinic. Clinical CDR can vary due to the inherent difficulty of grading (described above) and physician subjectivity [9,13,14,15,16,17,18,19,20]. As expected, our results showed a moderate correlation between grader and clinic CDR values at the vertical meridian. This finding is notable, as it demonstrates an advantage over automated imaging methods (HD-OCT and HRT), which can be limited by poor correlation with clinical grading [28]. However, similar to these instruments, our study did show lower correlation of grader and clinical CDRs in optic nerves with extreme CDR values (i.e., very low or high). 

We also tested the genetic association of three SNPs, which were previously associated with clinical VCDR in our GWAS, with grader VCDR measures. One SNP that was located in the *MPDZ* gene was significantly associated with grader VCDR and grader CDR area ratio. The *MPDZ* gene encodes a multiple PDZ domain crumbs cell polarity complex protein. Novel variants in this gene were previously reported in a multi-ethnic Asian GWAS study as associated with central corneal thickness, but not with CDR [37]. We hypothesize that the other two SNPs did not reach significance due to the smaller size of the cohort for grader VCDR (*n* = 623) versus the larger GWAS (*n* = 5049). Overall, however, these results suggest that grader CDR values, which provide more robust, quantitative, and precise CDR measures, can be used in future studies to associate distinct phenotypes with genetic variation. 

The inclusion of trained graders to outline the cup and disc, rather than automated algorithms, provides our approach with several advantages. This method was previously shown to be reproducible and to have high inter-grader reliability [22]. Many other algorithms use automated methods to locate and segment the optic cup and disc boundaries, such as ellipse fitting to smooth boundaries [25], edge detection with the Circular Hough Transform [31,32], or pixel classification [29,30]. These techniques are typically quicker, but they may not precisely capture the more irregular features or boundaries of discs, due to challenges such as the interference of blood vessels or confounding differences in illumination. More recently, one group developed an algorithm that calculated CDR values at 15-degree intervals of the cup and disc and fitted a probabilistic spatial model to the profiles [45]. This algorithm can estimate the probability of glaucoma given a certain profile with high accuracy. It uses monoscopic images and a semi-automatic (grader-marked clinical landmarks along the cup and disc boundary) or automatic method of segmentation, with the shape ultimately approximated by two ellipses. Similarly to our study, this approach was able to successfully distinguish between glaucoma and control patients and showed healthy and glaucomatous discs have differences at all degree locations [45]. 

Limitations of our study include the training of non-physician graders to obtain the outlines of cup and disc boundaries. Though this step increases accuracy, as discussed above, the training requires a significant amount of time. Our system was also challenging to compare with clinical grading, as clinical CDRs on patients in our cohort were solely obtained at the vertical meridian. Thus, we were only able to obtain correlations between grader and clinical values at the vertical bisector. Finally, the number of patients included in the genetic validation using grader VCDR was limited, because we only included glaucoma cases with both clinical and grader VCDR values. This may have hindered the validation of two SNPs from the POAAGG GWAS, which had a significantly larger cohort (5049 patients versus 623 patients).

At the current time, we envision our method being used in glaucoma research studies. The system can accurately capture the full morphology of the optic nerve at all locations, rather than just at the vertical meridian. This information can be used to examine a number of indices, such as cup centricity or eccentricity, rim thinning, and notching. This approach also has the potential to enable better tracking of glaucoma progression longitudinally, as nuances of increased optic nerve cupping will be easier to identify over time. 

Finally, we intend to use these data to examine the association of genetic variants with optic nerve morphology in the POAAGG population. This precision phenotyping may allow for the discovery of genetic risk factors that help to define subtypes of POAG. We plan to investigate the association of these subtypes with different features of ON morphology defined by our algorithm, such as notching and eccentric/symmetric cupping. Our appreciation of these distinct phenotypes in association with different genotypes will allow us to develop individualized screening and therapeutic modalities. We also aim to develop a polygenic risk model, using the 360 degree CDR values. These applications could lead to future improved screening and individualized treatments based on better understanding of distinct underlying genetic predispositions. 

## Figures and Tables

**Table 1:** Association between SNPs previously associated with clinical VCDR with grader CDR measurements in glaucoma cases (N = 623 patients, 1222 eyes).

			Grader Cup-to-Disc Area Ratio	Grader Vertical Cup-to-Disc Ratio
SNP (rs ID)	Number of Risk Alleles	# of Eyes	Adjusted Mean (SE) *	Adjusted Mean (SE) *
2:237653539_G (rs12328841)	0	458	0.50 (0.01)	0.69 (0.01)
	1	584	0.50 (0.01)	0.69 (0.01)
	2	180	0.47 (0.01)	0.66 (0.01)
	*p*-value ^§^		0.17	0.08
9:13173885_G (rs4740546)	0	1169	0.49 (0.01)	0.68 (0.00)
	1	53	0.55 (0.02)	0.73 (0.02)
	*p*-value ^§^		0.02	0.01
10:127738557_G (rs ID-N/A)	0	406	0.49 (0.01)	0.68 (0.01)
	1	582	0.49 (0.01)	0.68 (0.01)
	2	234	0.49 (0.01)	0.68 (0.01)
	*p*-value ^§^		0.65	0.82

* Adjusted by age and gender. # = number. SE = standard error. ^§^ From test of linear trend by model, the number of risk allele as a continuous variable. The inter-eye correlation is accounted for by using the generalized estimating equation.

## Data Availability

The genotypic data of subjects described in this study are openly available from the database of Genotypes and Phenotypes (dbGAP), under accession phs001312. Due to the nature of this research, the participants of this study did not agree for their stereo disc images to be shared publicly, so these data are not available publicly.

## Supplementary Materials

The following are available online at https://www.mdpi.com/article/10.3390/genes12121961/s1. Figure S1: Mean (orange) and standard deviation (blue) of the CDR at each degree location of stereo disc photos, for patients in Bins 4, 6, and 8. Figure S2: Grader outlines from stereo disc images, averaged and converted into polar coordinates, for eyes in Bins 4, 6, and 8. Figure S3: Plot of the mean (and standard deviation) of the +/− 50 degrees surrounding the maximum half cut-through CDR location for patients from Bins 4–8 with (N = 82) and without (N = 805) notches in their cup contours.
