# Supplementary material for: A Precise Method to Evaluate 360 Degree Measures of Optic Cup and Disc Morphology in an African American Cohort and Its Genetic Applications
Source: Genes (Basel). 2021 Dec 9;12(12):1961. doi: 10.3390/genes12121961 (PMC8701339; doi:10.3390/genes12121961)
Supplement: Supplementary file 1 [file genes-12-01961-s001.zip › genes-1488086-supplementary.pdf]

Supplementary Information

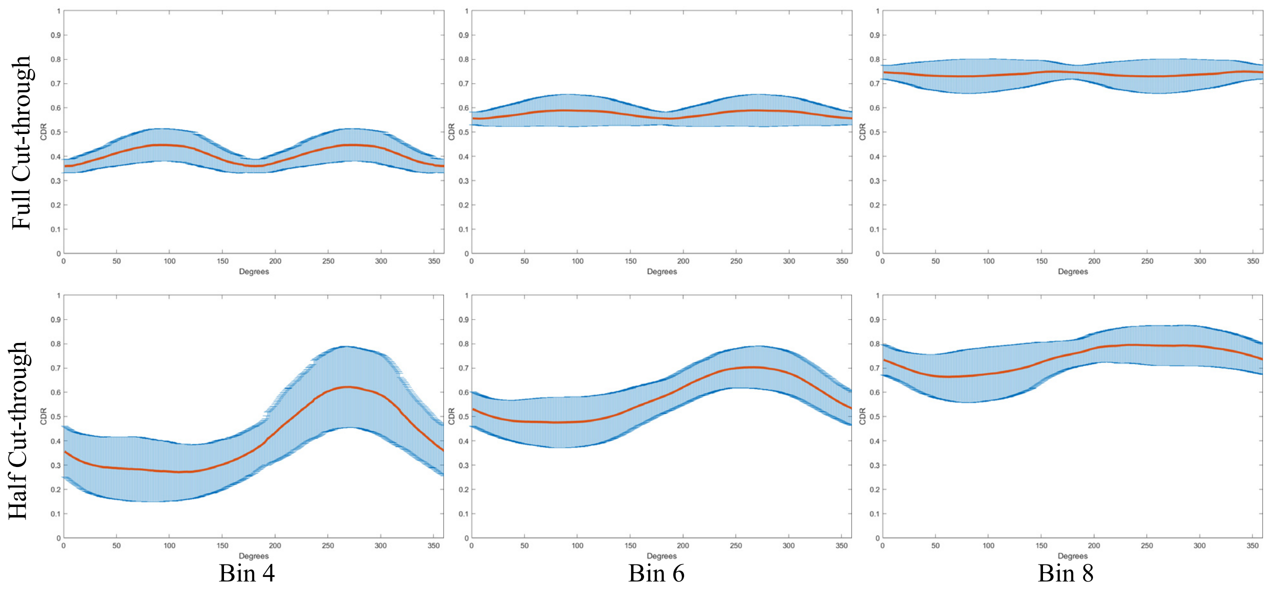

Figure S1: Mean (orange) and standard deviation (blue) of the CDR at each degree location of stereo disc photos, for patients in Bins 4, 6, and 8. The top row shows the full cut-through measurements and the bottom row shows the half cut-through measurements. Measurements include both OD and OS eyes from each bin, with the measurements from the OS eyes horizontally inverted to match the OD nasal/temporal directions.

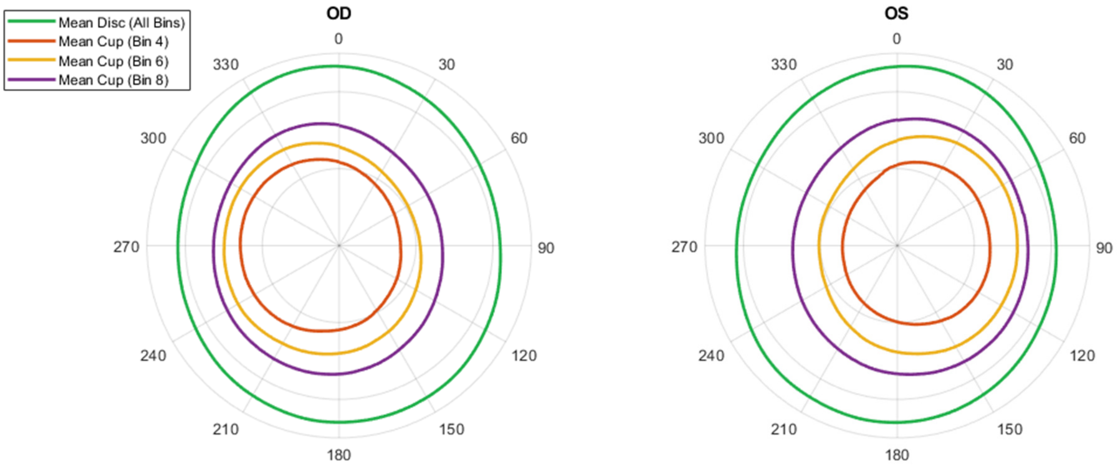

Figure S2: Grader outlines from stereo disc images, averaged and converted into polar coordinates, for eyes in Bins 4, 6 and 8. The means of the cup (red, orange, purple) and disc (green) contours outlined by two graders on each stereo disc photo at three CDR bins (Bin 4 [0.3 <= CDR < 0.4], Bin 6 [0.5 <= CDR < 0.6], and Bin 8 [0.7 <= CDR < 0.8]) and separated by eye. The outlines were binned according to clinically measured CDR for each subject. The polar origin for each individual drawing was moved to the centroid of its disc.

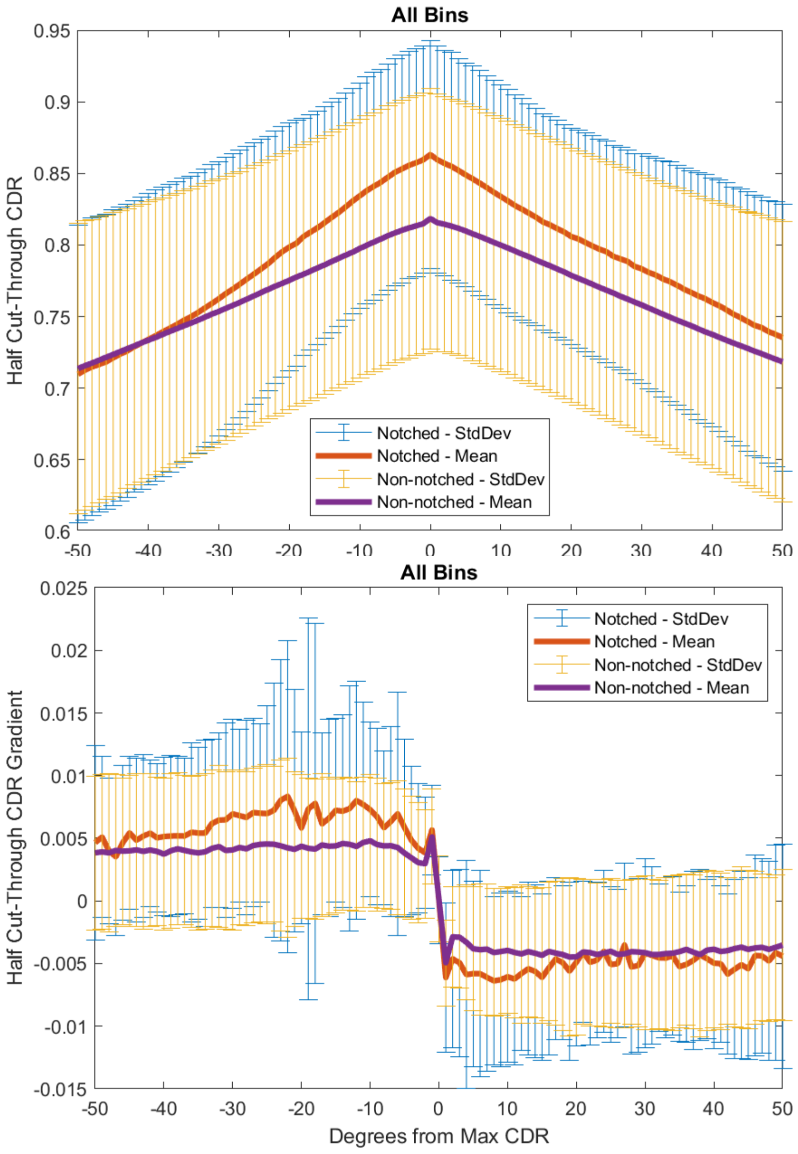

Figure S3: Plot of the mean (and standard deviation) of the +/- 50 degrees surrounding the maximum half cut-through CDR location, for patients from Bins 4-8 with (N=82) and without (N=805) notches in their cup contours. Top shows the half cut-through CDR values at each degree, and bottom shows the gradient of the half cut-through CDR at each degree. Measurements include both OD and OS eyes from each bin, with the measurements from the OS eyes horizontally inverted to match the OD nasal/temporal directions.
